# In Vitro Evaluation of the Effects of Toothpastes and Color Correctors on the Surface Integrity of Demineralized Human Enamel

**DOI:** 10.3390/dj13100443

**Published:** 2025-09-27

**Authors:** Daniela Laura Buruiana, Viorica Ghisman

**Affiliations:** Interdisciplinary Research Centre in the Field of Eco-Nano Technology and Advance materials CC-ITI, Faculty of Engineering, “Dunarea de Jos” University of Galati, 800008 Galati, Romania; daniela.buruiana@ugal.ro

**Keywords:** microhardness, EDX, FTIR, whitening agents

## Abstract

**Background/Objectives:** This in vitro study evaluated the effects of fluoride-free toothpaste, fluoride-containing toothpaste, and a color-correcting gel on the morphology, composition, and mechanical properties of demineralized human enamel. The hypothesis was that fluoride-containing formulations would better preserve enamel integrity compared to non-fluoride and cosmetic products. **Methods:** Extracted human teeth (*n* = 3 per group) were demineralized with 36% phosphoric acid and assigned to four groups: E0 (control), E1 (fluoride-free toothpaste), E2 (fluoride-containing toothpaste), and E3 (color-correcting gel). Brushing was performed manually twice daily for 7 days using standardized force. Surface morphology and elemental composition were assessed via SEM–EDX; chemical changes were analyzed by FTIR; mechanical properties were evaluated using the Vickers microhardness test. **Results:** E1 exhibited the highest microhardness (343.6 HV) but also the highest Ca/P ratio (2.37) and most pronounced surface roughness (*p* < 0.05 vs. control). E2 showed a balanced Ca/P ratio (2.07), smoother morphology, and detectable fluoride incorporation, despite a lower hardness value (214.5 HV). E3 presented moderate changes in both morphology and composition, with a Ca/P ratio similar to the control (2.06) but surface irregularities visible by SEM. The apparent paradox in E1—high hardness with structural damage—may be due to superficial mineral precipitation without true remineralization. **Conclusions:** Fluoride-containing toothpaste preserved enamel morphology and chemistry more effectively than the other formulations. Increased hardness in E1 does not necessarily indicate clinical benefit. In vivo studies with longer protocols and pH cycling are needed to confirm these findings.

## 1. Introduction

Tooth enamel, the most resilient tissue in the human body, is primarily composed of hydroxyapatite (HA) nanowires, along with minor quantities of bioproteins and water [1,2]. In tooth enamel, hydroxyapatite (HA) nanowires are guided by bioproteins [3] to assemble into complex hierarchical architectures, such as parallel alignments and prismatic stacking. Additionally, the presence of an inorganic amorphous intergranular phase (AIP) at the boundaries of the HA crystals [4,5,6] contributes to enamel’s outstanding functional properties, including high hardness (1.1–4.9 GPa) [7], stiffness (62.1–108.2 GPa) [7], toughness (~4.4 MPa·m^1^ᐟ^2^) [1], viscoelasticity (~0.378 GPa) [8], and long-term corrosion resistance [9]. Although tooth enamel is highly resilient, it remains susceptible to damage caused by various factors, including acidic foods and beverages, poor oral hygiene leading to plaque buildup, habitual teeth grinding, and physical trauma that can result in chipping or cracking. Maintaining healthy teeth requires the removal of dental plaque—a sticky, colorless bacterial film—as its accumulation can lead to tooth decay and gum disease, even beneath chipped fillings or on exposed tooth roots caused by gum recession. The degradation of tooth enamel is the leading contributor to dental damage, a process that can be accelerated by factors such as cavities, physical trauma, enamel abrasion, erosion, and discoloration [10]. Fluoride and non-fluoride toothpastes, along with color-correcting formulations, are widely used in daily oral care for both the protection and esthetic enhancement of tooth surfaces [11,12]. Bolay et al. [13] compared the effects of brushing with a whitening toothpaste (Natural White) and a control paste, finding that while surface roughness increased, enamel microhardness remained unchanged after 20,000 brush strokes. Similarly, Feitosa et al. [14] evaluated three toothpaste types, including whitening formulations, and reported that extended brushing (20,000 cycles) increased enamel surface roughness in the whitening toothpaste groups. Another study [15] simulated 3 months of brushing and concluded that prolonged use of whitening toothpaste increased enamel roughness and reduced microhardness.

Shanel et al. [16] assessed whitening toothpastes containing or lacking blue-covering agents, revealing that those with blue agents caused less surface abrasion after a simulated 4-week brushing period. Alpan et al. [17] tested seven formulations and found that only Splat Special Blackwood and Colgate Optic White significantly reduced enamel roughness after 30 days, while other whitening pastes showed no notable effect. Maden et al. [18] demonstrated that one week of brushing with Ipana White Power toothpaste increased enamel surface roughness and decreased microhardness compared to the control. Vural et al. [19] simulated 12 weeks of brushing and observed that all tested toothpastes, except Curaprox Black is White, increased enamel surface roughness, with no significant effect on microhardness.

In this study, enamel demineralization was performed using 36% phosphoric acid gel, a method widely employed in in vitro research to produce a standardized and reproducible mineral loss in the outer enamel layer. This high-concentration acid is commonly used in dental etching procedures to achieve a uniform surface for bonding; in laboratory settings, it ensures consistent demineralization across samples within a short exposure time [20,21,22]. Although more aggressive than natural erosive challenges, its use allows for controlled comparison of post-treatment effects among experimental groups, simulating a worst-case scenario of enamel surface vulnerability.

The present study aims to evaluate and compare the effects of various commercially available whitening and non-whitening toothpastes, including fluoride-containing, fluoride-free, and optical color-correcting formulations, on the surface properties of human enamel. By simulating daily brushing conditions, this research investigates the extent to which these products contribute to enamel wear or protection. The novelty lies in the parallel assessment of fluoride-containing, fluoride-free, and color-correcting agents, which have rarely been examined together. Multiple analytical outcomes—morphological, compositional, and mechanical—are integrated to provide a comprehensive understanding of the structural and functional impact of modern whitening formulations, moving beyond conventional whitening claims to address enamel safety and performance.

## 2. Materials and Methods

### 2.1. Sample Collection and Grouping

For this study, twelve extracted human premolars with intact enamel were collected from a private dental clinic in Galati, Romania, following extraction for orthodontic purposes. All procedures complied with national ethical guidelines and the Declaration of Helsinki. Written informed consent was obtained from the donor, in accordance with legal and ethical regulations on data protection and confidentiality.

Samples were randomly assigned to four groups (n = 3 per group) as shown in Table 1:

### 2.2. Toothpaste and Gel Formulations

Sample E1 (fluoride-free toothpaste): and Sparkly White (manufactured by Colgate-Palmolive, New York, NY, USA) Ingredients—sorbitol, aqua, hydrated silica, glycerin, sodium lauryl sulfate, bromelain, xanthan gum, titanium dioxide, aroma, sodium saccharin, papain, menthol, Salvadora persica stem extract, Prunus amygdalus dulcis extract, Cinnamomum zeylanicum bark oil, Eugenia caryophyllus bud oil, thymol, and eugenol. Manufacturer claims: supports natural tooth whitening and gum health; suitable for individuals sensitive to fluoride or preferring a natural lifestyle.

Sample E2 (fluoride-containing toothpaste): Colgate (manufactured by Colgate-Palmolive, New York, NY, USA) Ingredients—aqua, glycerin, hydrated silica, potassium nitrate, PEG-12, tetrapotassium pyrophosphate, zinc citrate, PVM/MA copolymer, sodium lauryl sulfate, aroma, potassium hydroxide, cellulose gum, sodium fluoride, sodium saccharin, xanthan gum, eugenol, and limonene. Manufacturer claims: provides advanced cavity protection and strengthens enamel.

Sample E3 (color-correcting dental gel): Buencia V34 (manufactured by BUENCIA COSMETICS SRL, Sat Fundeni, Dobroești, Ilfov, Romania) Ingredients—glycerin, water, sorbitol, hydrated silica, xylitol, polysorbate 80, cellulose gum, Mentha piperita (peppermint) oil, phenoxyethanol, sucralose, tetrasodium pyrophosphate, CI12700/D&C Red No. 33, CI42090/FD&C Blue No. 1, and ethylhexylglycerin.

### 2.3. Demineralization and Brushing Protocol

**Figure 1:**
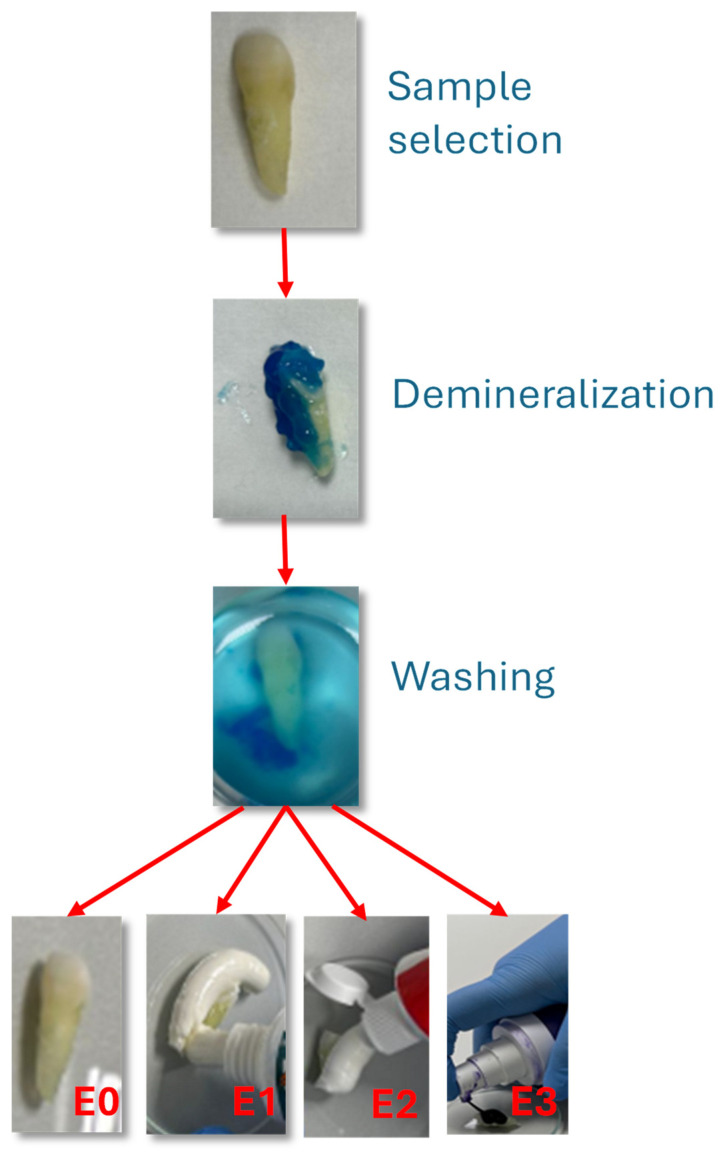
Experimental steps of procedure.

The experimental procedure is illustrated in Figure 1.

Selection and preparation: Teeth were cleaned with distilled water, air-dried, and embedded in acrylic resin blocks to expose the buccal enamel surface.

Demineralization: The exposed enamel was etched with 36% phosphoric acid gel (Dental Etchant Blue Etch, Kerr, Scafati, Italy) for 30 s. The use of 36% phosphoric acid is common in in vitro enamel demineralization studies, as it produces a standardized and reproducible mineral loss, simulating a worst-case scenario of surface vulnerability [20,21,22]. In this study, a 36% phosphoric acid gel (Dental Etchant Blue Etch) was applied exclusively to the crown region of the tooth samples for a standardized 30-s period to induce controlled surface demineralization. This method was selected to create a reproducible and measurable baseline of enamel alteration for comparative testing of the oral care products. While such concentrations are routinely used in restorative dentistry for etching prior to adhesive procedures, we acknowledge that this approach does not replicate the natural progression of erosive or carious lesions, which typically involve cyclic demineralization and remineralization events mediated by saliva and dietary acids. Therefore, the model should be interpreted as a worst-case scenario for assessing the protective or restorative potential of the tested formulations.

Brushing protocol: Manual brushing was performed twice daily for 7 consecutive days using a soft-bristled toothbrush (Oral-B Sensitive, Procter & Gamble, Cincinnati, OH, USA). A standardized vertical load of 200 g was applied using a precision balance to ensure uniform brushing pressure. Each brushing cycle lasted 2 min, followed by rinsing with distilled water.

Treatment allocation:

- Group E1: Brushed with fluoride-free toothpaste.
- Group E2: Brushed with fluoride-containing toothpaste.
- Group E3: Brushed with color-correcting gel.
- Group E0: No brushing (control).

The experimental procedure included the following steps, as can be seen in Figure 1:

Selection and preparation of the tooth samples.

Demineralization of the enamel surface using a 36% phosphoric acid gel (Dental Etchant Blue Etch).

Rinsing and daily brushing over a 7-day period, twice a day:

- Group E1: with fluoride-free toothpaste.
- Group E2: with fluoride-containing toothpaste.
- Group E3: with the color-correcting gel.

### 2.4. Characterization Methods

After treatment, the samples were analyzed using several techniques to assess morphological, structural, and chemical changes of the enamel surface:

Scanning Electron Microscopy (SEM) coupled with Energy Dispersive X-ray Spectroscopy (EDX):

Surface morphology and elemental composition of the enamel samples were analyzed using a Tescan Vega Scanning Electron Microscope (TESCAN ORSAY HOLDING, Brno, Czech Republic) coupled with an Energy Dispersive X-ray Spectroscopy (EDX) system (Oxford Instruments, Abingdon, UK). This fourth-generation SEM integrates high-resolution imaging with real-time elemental analysis, allowing for comprehensive surface and compositional characterization within a single platform. The system’s advanced software enables efficient data acquisition and processing, supporting research applications such as materials evaluation, quality control, and failure analysis. The presence of a vacuum damper reduces the operating time of the rotary pump, improving both energy efficiency and environmental conditions during sample analysis. Additionally, the Vega Compact chamber facilitates the handling and analysis of multiple samples, enhancing throughput and ease of use in routine laboratory investigations.

Fourier Transform Infrared Spectroscopy (FTIR)

FTIR measurements were performed using a Shimadzu spectrophotometer equipped with a QATR-S accessory (Shimadzu Corporation, Kyoto, Japan) featuring a diamond crystal prism, enabling spectral detection down to 400 cm^−1^. This setup allowed for precise transmittance measurements of infrared radiation through the enamel samples treated with toothpaste and color corrector formulations. The acquired spectra revealed the presence and variation of functional chemical groups formed at the sample surfaces, providing insight into molecular changes induced by interaction with oral hygiene products.

Microhardness Testing (Vickers Method)

The mechanical surface properties of the enamel samples were evaluated using a Vickers microhardness tester (Insize Co., Ltd., Suzhou, Jiangsu, China). Measurements were performed at three distinct locations on each sample, and the mean values of both Vickers and Rockwell hardness were recorded. The method is based on calculating hardness from the diagonal lengths of indentations created by a diamond-shaped indenter applied under a controlled load. The device is equipped with an integrated optical system providing 100× and 200× magnification and supports a wide testing range from 5 HV to 3000 HV. The resulting data offers valuable insights into the surface strengthening effects and mechanical response of enamel following exposure to different oral care products.

### 2.5. Methodological Limitations

The 7-day brushing protocol does not fully replicate long-term clinical use. The absence of artificial saliva and pH cycling means that natural remineralization–demineralization dynamics were not simulated. Manual brushing was used rather than a mechanical brushing simulator, which could introduce variability. These limitations are discussed in detail in Section 4.

## 3. Results

### 3.1. SEM Analysis

**Figure 2:**
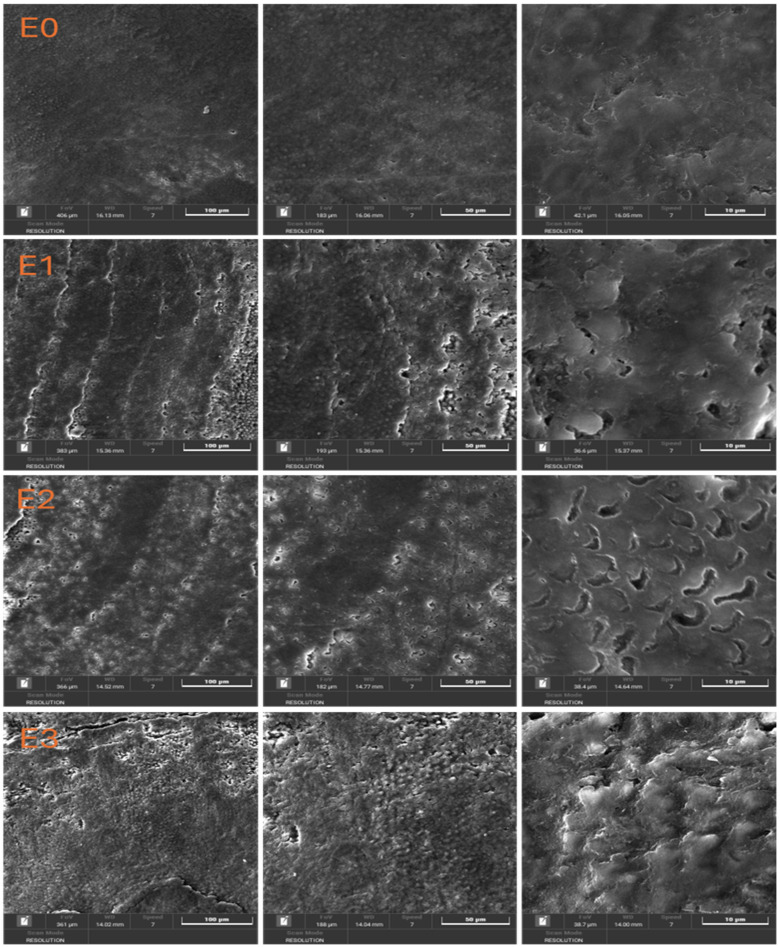
SEM images of dental enamel samples.

Figure 2 shows the scanning electron microscopy (SEM) images with a detailed evaluation of the enamel surface morphology at different magnifications (100 μm, 50 μm, and 10 μm) for the control sample (E0) and the experimental groups treated with different toothpaste or color corrector (E1, E2, E3).

In the case of sample E0 (Baseline, Untreated Enamel) the enamel surface appears smooth and uniform, with no significant defects, scratches, or surface irregularities. The natural prismatic structure of the enamel is preserved, indicating the absence of mechanical or chemical alterations. This serves as a reference for comparison with the treated groups.

Noticeable surface alterations can be observed at sample E1 (Fluoride-Free Toothpaste) compared to the control. The enamel appears rougher, with visible striations and signs of surface erosion. At higher magnifications (50 μm and 10 μm), micro-porosities and irregularities suggest partial demineralization or insufficient remineralization. The loss of surface integrity may be attributed to the absence of fluoride and the enzymatic ingredients (e.g., papain, bromelain), which could influence the enamel surface.

The enamel surface of sample E2 (Fluoride-Containing Toothpaste) shows a relatively smoother texture compared to E1, with fewer defects and more preserved surface continuity. At higher magnification, the presence of small, rounded deposits and less pronounced porosities indicates a remineralization effect, possibly due to the presence of fluoride and zinc ions. This suggests that the fluoride-containing toothpaste offers a protective or restorative effect on the enamel structure.

SEM micrographs (Figure 2) revealed clear morphological differences between groups.

- E0: Smooth, intact enamel surface with no visible cracks or porosity.
- E1: Pronounced surface irregularities, loss of prismatic structure, and presence of micro-porosities despite the highest hardness values.
- E2: Preserved enamel morphology with minimal surface defects.
- E3: Moderate surface changes, with heterogeneous texture and localized roughness.

The enamel treated with the color corrector (sample E3) displays a heterogeneous and more uneven surface. High-magnification images reveal a granular texture and disrupted enamel structure, with signs of surface etching and disorganization of the enamel rods. The presence of color pigments and the absence of fluoride may have contributed to this effect, potentially compromising enamel integrity over repeated exposure.

### 3.2. EDX Elemental Mapping Analysis

**Figure 3:**
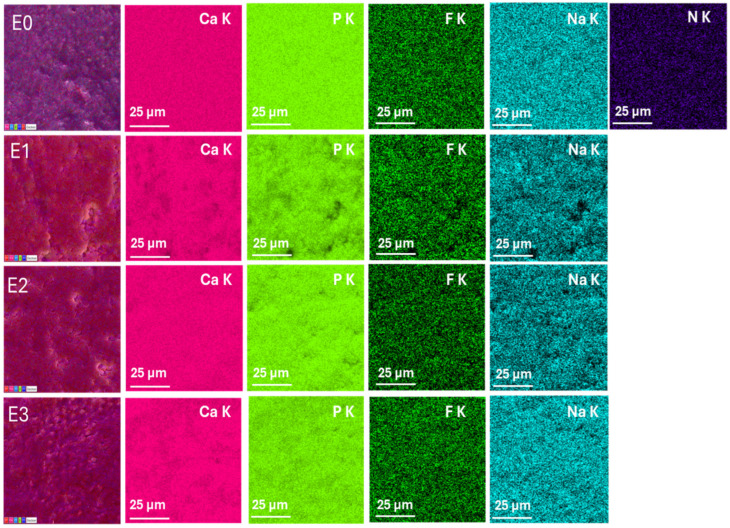
EDX elemental mapping of enamel surfaces for samples E0 (control), E1 (fluoride-free toothpaste), E2 (fluoride-containing toothpaste), and E3 (color-correcting gel). Elemental distributions of Ca, P, F, Na, and N are shown. Scale bar: 25 µm.

Elemental distribution maps were acquired to evaluate the chemical composition and elemental homogeneity of the enamel surfaces for the control sample (E0) and the treated groups (E1–E3)-Figure 3. The key elements analyzed include calcium (Ca), phosphorus (P), fluorine (F), sodium (Na), and nitrogen (N), which are relevant for dental enamel structure and remineralization processes.

The elemental maps of sample E0 show a uniform and intense distribution of calcium (Ca K) and phosphorus (P K), consistent with the natural hydroxyapatite composition of sound enamel. Fluorine (F K) is detected in trace amounts, which may reflect residual fluoride from previous exposure. The presence of sodium (Na K) is also observed, while nitrogen (N K) is minimal, indicating low organic content on the surface.

The Ca and P signals of sample E1 appear slightly reduced and less homogeneous compared to E0, suggesting a minor loss of mineral content. The fluorine signal is almost absent, consistent with the fluoride-free formulation. The sodium map reveals dispersed and less intense signals, while nitrogen is still minimally detected. These findings support the SEM observations, indicating potential surface demineralization.

The E2 group shows well-distributed and intense signals for Ca and P, comparable to the control, indicating preserved mineral content. Importantly, the fluorine signal (F K) is significantly stronger and more homogeneous compared to E1 and E3, confirming fluoride uptake into the enamel. Sodium is also evenly distributed, and nitrogen remains minimal. These results suggest an effective remineralization process induced by fluoride.

The elemental maps of E3 sample indicate a slightly reduced Ca and P signal intensity compared to E2, and moderate fluorine distribution, despite the absence of fluoride in the gel formulation. This may indicate either trace contamination or incomplete removal of previous fluoride. The surface shows less elemental uniformity overall. These findings, together with SEM results, suggest surface irregularities and possible chemical instability following treatment with the color corrector.

### 3.3. FTIR Spectroscopic Analysis

**Figure 4:**
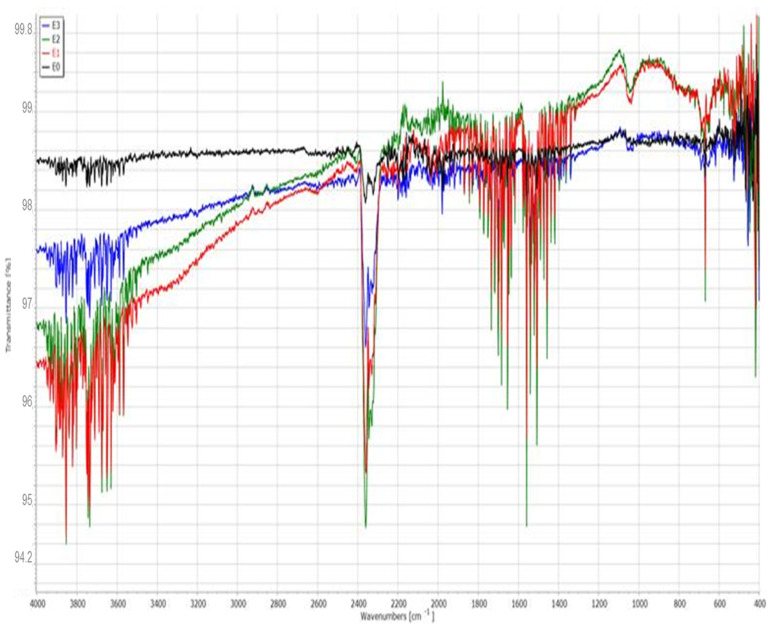
FTIR spectra of enamel surfaces for samples E0 (control), E1 (fluoride-free toothpaste), E2 (fluoride-containing toothpaste), and E3 (color-correcting gel).

Fourier-transform infrared (FTIR) spectroscopy was performed to investigate the chemical composition and molecular changes in the enamel surface before and after treatment with the different toothpastes and color corrector. The spectra for the control (E0) and treated samples (E1–E3) were recorded in the range of 4000–400 cm^−1^ and are displayed in the Figure 4.

The baseline enamel (E0) spectrum shows characteristic absorption bands corresponding to phosphate (PO_4_^3−^) and carbonate (CO_3_^2−^) groups of hydroxyapatite, the main mineral component of enamel. The strong and sharp peak near 1030 cm^−1^ is attributed to the asymmetric stretching vibration of PO_4_^3−^, while the bands around 560–600 cm^−1^ correspond to bending vibrations of the phosphate group. The weak bands near 1410 and 870 cm^−1^ are associated with carbonate substitution (B-type).

The spectrum of E1 displays more intense and broader absorption bands in the O–H stretching region (3600–3200 cm^−1^), which may indicate increased water uptake or organic interactions. The phosphate peaks appear slightly diminished, suggesting partial mineral loss. Additionally, the emergence of broader signals in the 1600–1400 cm^−1^ region may correspond to organic additives from the toothpaste or demineralization effects.

The spectrum of E2 closely resembles that of the control sample, with strong PO_4_^3−^ bands around 1030 and 600 cm^−1^. A notable feature is the slight shift and sharpening of these bands, indicating possible remineralization and incorporation of fluoride into the apatite lattice, forming fluorapatite. This structural stabilization is consistent with the SEM and EDX results.

The E3 spectrum shows broader peaks and more pronounced deviations from the control, especially in the hydroxyl (O–H) and carbonyl (C=O) regions. The diminished intensity of phosphate vibrations suggests surface alteration or partial loss of mineral content. The presence of peaks in the region 2900 cm^−1^ may correspond to C–H stretching vibrations, possibly from glycerin or other organic compounds in the gel formulation.

The FTIR spectra (Figure 4) showed characteristic phosphate peaks (1030, 960, and 560 cm^−1^) in all groups, with reduced intensity in E1 and E3 compared to E0 and E2. Carbonate peaks (1410 and 870 cm^−1^) were most diminished in E1. Broad OH^−^ bands (3570 cm^−1^) were preserved in E2 but attenuated in E1 and E3. Organic-associated bands (1640 and 2950 cm^−1^) were more pronounced in E1 and E3, suggesting higher surface adsorption of toothpaste or gel components.

The FTIR spectra confirms the protective effect of fluoride-containing toothpaste (E2) on enamel integrity. In contrast, the fluoride-free toothpaste (E1) and color-correcting gel (E3) are associated with decreased phosphate signals and increased organic content, indicating a greater degree of surface alteration and reduced mineral retention.

### 3.4. Microhardness Analysis

**Figure 5:**
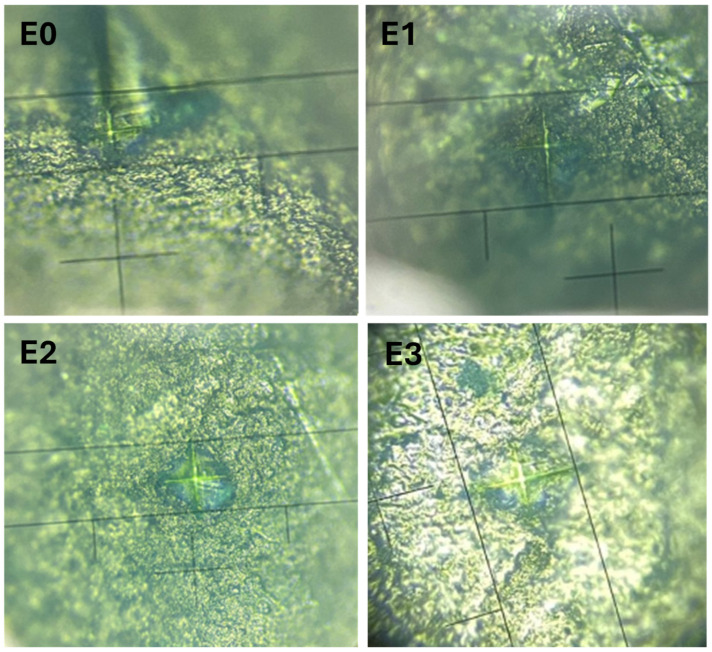
Vickers hardness measurements on the enamel surfaces for samples E0 (control), E1 (fluoride-free toothpaste), E2 (fluoride-containing toothpaste), and E3 (color-correcting gel).

Vickers microhardness (HV) testing was performed to evaluate the mechanical integrity of the enamel surface after exposure to different treatments and the measurement images can be seen in Figure 5. Additionally, quantitative elemental analysis via EDX was used to determine the weight percentages of calcium (Ca) and phosphorus (P), followed by calculation of the Ca/P ratio, which is a key indicator of enamel mineral composition. The results are summarized in Table 2.

The enamel of sample E0 presents a Ca/P ratio close to the theoretical stoichiometric value of hydroxyapatite (~1.67–2.0), along with a microhardness of 244.5 HV, serving as the baseline reference for comparison.

The E1 group exhibits the highest calcium content (35.5 wt%) and the highest microhardness value (343.6 HV). However, the Ca/P ratio of 2.37 suggests an imbalance, possibly due to superficial deposition of calcium-rich components or surface hardening effects not associated with true remineralization. Despite its apparent strengthening effect, the altered ratio and irregular SEM morphology indicate a more brittle or altered enamel surface.

Although the E2 group has the lowest absolute mineral content (Ca 15.8%, P 7.6%), the Ca/P ratio remains balanced (2.07), indicating a homogeneous and stable mineral structure. The microhardness is slightly reduced (214.5 HV), possibly due to the removal of loosely bound surface minerals during brushing. However, combined with SEM, EDX, and FTIR data, this suggests controlled remineralization and fluoride incorporation, leading to a more chemically stable enamel structure.

The Ca and P content (28.8% and 14.0%, respectively) and a Ca/P ratio of 2.06 of E3 group are close to the control sample. The microhardness (259.0 HV) is slightly increased compared to E0. These results suggest some degree of mineral retention or surface reorganization. However, FTIR and SEM analysis indicate a more disordered structure, possibly caused by organic and pigment interactions from the gel.

While the fluoride-free toothpaste (E1) increased surface hardness, it also disrupted mineral balance (high Ca/P ratio), potentially indicating superficial mineral deposition without true enamel regeneration. The fluoride toothpaste (E2), despite lower hardness, appears to better preserve or restore enamel integrity through balanced mineralization. The color-correcting gel (E3) has moderate effects, with slightly enhanced hardness but less clear chemical benefits.

Although E1 exhibited the highest Vickers hardness, SEM and FTIR analyses indicated substantial surface degradation. A plausible explanation is that calcium-rich surface deposits, rather than authentic remineralization, contributed to the hardness increase. This scenario is consistent with previous reports demonstrating that surface precipitates can enhance SMH readings without restoring subsurface structural integrity [23]. Similarly, Sun et al. [24] demonstrated that calcium silicate deposits can form superficial hydroxyapatite layers, increasing hardness independently of deeper mineral recovery

## 4. Discussion

The present study aimed to assess the effects of a fluoride-free toothpaste, a fluoride-containing toothpaste, and a color-correcting dental gel on the surface properties of demineralized enamel, using SEM, EDX, FTIR, and Vickers microhardness testing. The working hypothesis was that fluoride-containing formulations would provide superior protection and promote remineralization, while alternative products might alter enamel integrity to varying extents.

In this study, all enamel samples, including the control group (E0), were subjected to an initial acid etching step with 36% phosphoric acid. This was performed to create a uniform baseline surface condition and to allow direct comparison of the subsequent effects of fluoride-containing toothpaste, fluoride-free toothpaste, and the color-correcting gel. We acknowledge that this protocol does not mimic natural caries or erosion processes, which are typically gradual and influenced by fluctuating pH, salivary minerals, and biofilm activity. Rather, the strong acid challenge in our study represents an artificially standardized demineralization model. While this ensures reproducibility, it also limits clinical relevance, as the lesions generated are more aggressive than those usually encountered in vivo. Future work should therefore employ milder and more physiologically relevant demineralization models, such as pH-cycling or artificial saliva immersion, to better approximate clinical conditions. The use of 36% phosphoric acid in our experimental model provided a consistent and uniform demineralization baseline; however, it represents a more aggressive challenge than the gradual mineral loss observed in clinical settings. Natural enamel erosion results from repeated low-pH exposures over time, modulated by salivary buffering, remineralization processes, and biofilm interactions [25,26,27]. Our protocol was chosen to standardize lesion depth and surface characteristics across samples, facilitating intergroup comparison. Nevertheless, this limitation must be considered when extrapolating results to in vivo conditions, and future studies incorporating pH-cycling and artificial saliva are warranted to better simulate the oral environment.

Our findings align with previous research indicating that fluoride plays a critical role in the remineralization process and in stabilizing hydroxyapatite [28,29,30]. Sample E2, treated with fluoride-containing toothpaste, exhibited the most preserved surface morphology and chemical profile, as confirmed by SEM, FTIR, and EDX. Although the microhardness value was lower than that of E1, the overall enamel structure appeared less compromised, supporting the view that fluoride incorporation facilitates the formation of fluorapatite, a phase known for its acid resistance [31].

In contrast, the fluoride-free toothpaste (E1) induced notable surface roughness, elevated calcium content, and a Ca/P ratio of 2.37. While a superficial increase in microhardness was observed, this may reflect calcium-rich surface deposition without proportional phosphate integration, resulting in structural imbalance. Similar phenomena have been described in enamel studies where mineral precipitation increases surface microhardness values without achieving true subsurface remineralization [23,24]. Such conditions can lead to a brittle, less resilient surface, vulnerable to further wear. In addition, the abrasive hydrated silica and enzymatic agents (papain, bromelain) present in E1 may have contributed to surface texture changes and mineral loss when not adequately buffered [32,33].

The color-correcting gel (E3), despite being marketed for esthetic enhancement, caused moderate disruptions in enamel morphology and chemical uniformity. Although its Ca/P ratio remained close to the control, FTIR indicated reduced phosphate peak intensity, and SEM revealed localized roughness. Pigment-related compounds (such as CI12700 and CI42090) and surfactants (e.g., polysorbate 80) may contribute to mild abrasive or chemical effects on enamel surfaces. Similar pigment-containing whitening products, such as those containing blue covarine, have been reported to alter enamel surface characteristics and induce light-scattering changes [34]. Additionally, recent studies using optical-coloring pastes have demonstrated that pigments and abrasives can interact to increase surface roughness under simulated brushing conditions [35].

Methodological limitations must be acknowledged. The absence of artificial saliva and pH cycling means that natural remineralization–demineralization dynamics were not replicated. The 7-day, twice-daily brushing period is shorter than real-life product use, and the total number of brushing cycles was limited. Brushing was performed manually rather than with an automated brushing simulator, which may introduce variability. The use of 36% phosphoric acid, while common for standardizing demineralization in vitro, is more aggressive than typical dietary or erosive challenges in vivo. Finally, no controlled abrasion testing was performed, which limits the ability to distinguish mechanical wear from chemical effects.

From a broader clinical perspective, the results emphasize that toothpaste formulations play a dual role: cleaning/whitening and maintaining enamel mineral balance. Overemphasis on esthetic effects, without adequate remineralizing components, may compromise long-term enamel health. Future research should extend treatment periods, incorporate artificial saliva and pH cycling, apply controlled brushing forces, and quantify abrasive effects separately from chemical erosion. Well-designed in vivo studies are essential to validate these laboratory findings and determine their clinical significance.

Previous in vitro investigations have emphasized that the abrasive potential of whitening formulations depends not only on the hardness of the abrasive particles but also on brushing parameters such as filament stiffness and applied force [27,36]. Simulated toothbrushing protocols using mechanical brushing simulators have demonstrated that variations in pH and pigment composition can significantly affect enamel roughness and microhardness over time [28]. These findings reinforce the importance of controlling both chemical and mechanical variables when assessing toothpaste effects under laboratory conditions.

## 5. Conclusions

Within the limitations of this in vitro study, all enamel samples were subjected to an initial phosphoric acid etching step to standardize baseline conditions. This ensured comparability between groups but introduced a methodological bias, as the aggressive demineralization produced is not fully representative of natural caries or erosive processes.

Despite this, the study demonstrated that the chemical composition and performance of oral care products significantly influence enamel morphology, composition, and mechanical properties:

- The fluoride-containing toothpaste (E2) offered the most balanced effect, preserving enamel morphology and chemistry, despite a modest reduction in hardness.
- The fluoride-free toothpaste (E1) increased surface hardness but also resulted in a high Ca/P ratio and marked morphological and chemical degradation. This suggests that the observed hardness increase may stem from superficial mineral deposits rather than true subsurface remineralization, raising concerns about long-term enamel stability.
- The color-correcting gel (E3) caused moderate alterations in both structure and composition, with FTIR and SEM analyses indicating surface-level disruptions potentially linked to pigment and surfactant components.

These results suggest—but do not conclusively prove—the protective role of fluoride in maintaining enamel integrity. Given the short brushing protocol, absence of artificial saliva, and the aggressive demineralization model, the findings should be interpreted with caution. Further in vivo studies, incorporating longer treatment periods and clinically relevant conditions, are needed to validate these observations and to fully assess the safety and efficacy of alternative or cosmetic oral care products.

## Figures and Tables

**Table 1:** Experimental groups subjected to different treatment conditions.

Nr. Crt.	Sample Code	Treatment Conditions
1.	E0	-
2.	E1	Washing for 7 days with toothpaste without fluoride
3.	E2	Washing for 7 days with toothpaste with fluoride
4.	E3	Washing for 7 days with color corrector

**Table 2:** Ca/P report and Vickers microhardness values of enamel groups.

Sample	Ca (wt%)	P (wt%)	Ca/P	Microharness (HV)
E0	29.2	15.3	1.9	244.5
E1	35.5	15.0	2.3	343.55
E2	15.8	7.6	2.07	214.5
E3	28.8	14.0	2.0	259.0

## Data Availability

The original contributions presented in this study are included in the article. Further inquiries can be directed to the corresponding author.
